# *Prosopis juliflora* hydrothermal synthesis of high fluorescent carbon dots and its antibacterial and bioimaging applications

**DOI:** 10.1038/s41598-023-36033-3

**Published:** 2023-06-15

**Authors:** Nadarajan Prathap, Putrakumar Balla, Muthugoundar Subramanian Shivakumar, Govindasami Periyasami, Ponmurugan Karuppiah, Krishnaraj Ramasamy, Srinivasan Venkatesan

**Affiliations:** 1 Department of Environmental Science, School of Energy and Environmental Sciences, Periyar University, Salem, India; 2 Department of Chemical Engineering and Applied Chemistry, Chungnam National University, Daejeon, Republic of Korea; 3 Department of Biotechnology, School of Biosciences, Periyar University, Salem, India; 4 Department of Chemistry, College of Science, King Saud University, P.O. Box 2455, Riyadh, 11451 Saudi Arabia; 5 Department of Botany and Microbiology, College of Science, King Saud University, P.O. Box 2455, Riyadh, 11451 Saudi Arabia; 6 Department of Mechanical Engineering, College of Engineering and Technology, and Director Centre for Excellence in Indigenous Knowledge Innovative Technology Transfer and Entrepreneurship, Dambi Dollo University, Dembi Dollo, Ethiopia

**Keywords:** Antimicrobial responses, Nanoscience and technology

## Abstract

Carbon dots have stimulated the curiosity of biomedical researchers due to their unique properties, such as less toxicity and high biocompatibility. The synthesis of carbon dots for biomedical application is a core area in research. In the current research, an eco-friendly hydrothermal technique was employed to synthesize high fluorescent, plant-derived carbon dots from *Prosopis juliflora* leaves extract (PJ-CDs). The synthesized PJ-CDs were investigated by physicochemical evaluation instruments such as fluorescence spectroscopy, SEM, HR-TEM, EDX, XRD, FTIR, and UV-Vis. The UV-Vis absorption peaks obtained at 270 nm due to carbonyl functional groups shifts of n→π*. In addition, a quantum yield of 7.88 % is achieved. The synthesized PJ-CDs showing the presence of carious functional groups O–H, C–H, C=O, O–H, C–N and the obtained particles in spherical shape with an average size of 8 nm. The fluorescence PJ-CDs showed stability against various environmental factors such as a broad range of ionic strength and pH gradient. The antimicrobial activity of PJ-CDs was tested against a *Staphylococcus aureus*, and a *Escherichia coli*. The results suggest that the PJ-CDs could substantially inhibit the growth of *Staphylococcus aureus*. The findings also indicate that PJ-CDs are effective materials for bio-imaging in *Caenorhabditis elegans* and they can be also used for pharmaceutical applications.

## Introduction

Pathogen such as bacteria, fungi, viruses, and parasites cause several diseases every year^1,2^. Emergence of drug resistant pathogens hence the antibiotic resistance has quickly become one of the world's major life-threatening problems^3^. Evolution and speed of bacterial drug resistance has outpaced exploration and development of new antibiotics which are hindered due to high cost and technical difficulties^4^. This warrants an urgent need for production of novel and new alternative antibacterial drugs. The swift advancement of nanotechnology over the last several decades has resulted in the creation of promising antibacterial treatment alternatives^5^. Nanomaterials containing metal nanoparticles such as Cu-NPs, Ag-NPs, and Cu-Te NPs usually produce reactive oxygen species and followed by discharge the metal ions; thereby leading to antimicrobial activity^6^. However, in addition to the protein inactivation, enzymatic inhibition, oxidative stress, and DNA damage have all contributed to the presence of metal nanoparticles in non-target cells which can damage the healthy tissues and this is why the metal nanoparticles have limited clinical applicability^7^. Carbon is a common non-metallic element that is abundant in environment, and it appears in a variety of allotropic forms including amorphous carbon, graphite, and diamond^8^. Kroto et al. (1985), Water solubility and high fluorescence luminescent carbon dot were first reported in 2006^9^. The Carbon dots (CDs) are normally pseudo nano-materials having amorphous and nano-crystalline structure and consist sp^2^/sp^3^ carbon, oxygen/nitrogen-based groups, and also post -altered groups^10^. There are two types of CDs syntheses methods namely bottom up and top-down methods^11^. In traditional top-down approaches, carbon source components are initially converted into nano materials and followed by different methods including acid discharge, laser ablation, and oxidations which are used^12^. CDs are also prepared by diverse bottom-up methods namely pyrolysis, carbonization, hydrothermal and microwave assisted^13^. CD structure mainly depends on its synthesis method and is responsible for its varied luminescence properties^14^. In general, the CDs have a core shells-like made of base carbon core with functional groups attached^15^. The CDs carry carbon atoms and other molecule elements which is accountable for the function of carbon dots^16^. Scientists involve in the use of CDs, due to their advantages such as water solubility, cost effective, high conductivity, tunable photoluminescence, easy surface modification, high stability and high biocompatibility across the globe^17,18^. CDs have been ideally used in wide ranging applications like dye degradation^19–21^, bioimaging^22–24^ detection of light-emitting diodes^25,26^ sensing^27,28^ and catalysis^29,30^. Till date, there are several antibacterial actions of CDs which have been verified in large number of studies, like mechanical destruction, oxidative stress and reduction of bacterial metabolism^31^ and photo-catalysis^32,33^. Among them the reactive oxygen molecules trigger oxidative stress is the main mechanism. Which is got activated by light to create singlet molecular oxygen (O_2_) or superoxide anion (O_2_·) or to act as a catalase to oxidise other agents^34^.

Natural biomaterials are resourceful, economical, and eco-friendly feasible method of carbon quantum dots since they are biocompatible, renewable, and transform to the bio-waste into valuable products^35,36^. There are several investigations on the preparation of carbon related natural materials, including *Psidium guajava* leaves^37^, *Curcuma longa* leaves^38^, *Osmanthus* leaves^39^, milk vetch and tea leaves and others, which have recently been reported^40^. *Prosopis juliflora* is a world worst invasive plant in Asia, Africa and Australia. This plant is capable of displacing natural plants from their habitats^41^.Since this plant cannot provide proper shelter the bird fauna is affected. The region’s water table in severely reduced and nutritive elements from the soil are depleted^42,43^.

Use of fluorescent tags to visualize either eukaryotic or prokaryotic cells which exist becoming widespread across all disciplines^44^. Luminescent dyes have usually been employed to stain bacterial species, fungus, and plants cells. Anionic and Cationic dyes are similarly known as luminesce, and used their capability to bind to biological constituents of bacterial cells^45^. Acridine orange, ethidium bromide, and fluorescence isothiocyanate are some of the fluorochromes frequently used^46^. However, for a more sustainable future, it is critical to use a material which is biocompatible, minimal toxic effect, cheap, and environment friendly. Luminescent nanomaterials fulfil all the above said properties and considered possible rivals to standard fluorescent dye probes, for application in biological labelling, chemical sensing and other fields^47,48^. When compared to standard fluorescent dyes, light emitting nanomaterials, have specific nanomaterial and are quantum size effect, which several advantages such as low fluorescent intensity, limited stability, and quick photo bleaching^49^. In the present study we synthesised facile hydrothermal process of CDs using *Prosopis juliflora* leaf as carbon source. A synthesised *Prosopis juliflora* carbon dot was evaluated for antibacterial effect on against Gram positive *Staphylococcus aureus*, and a Gram negative *Escherichia coli*. The fluorescence property of synthesized CDs was applied for selective analysis of nematode as a bioimaging application.

## Materials and Methods

### Materials

The collection of plant leaf materials was done according to the International and National guidelines for the purpose^50^. Aerial parts of *Prosopis juliflora* leaves were collected in December 2021, at Periyar University campus, Salem, India (11.7188°N, 78.0779°E). The sample was stored in plastic cover and brought to the research lab within 48 hrs. The collected plant leafs were washed carefully with tap water and then soaking in double distilled water (DDW) for five times, and finally shade drying for 15 days. The dried plant leaves leaf was grounded into a fine powder with a mechanical mixer blender. Five gram of plant leaves powder was dispersed in 50 mL of DDW and placed on Teflon autoclave and heated at 180ºC for 5 hrs. After cooling down to room temperature the natural brown colour solution was passed through Whatman No 1 filter paper and then centrifuged at 8000 rpm for 15 min to remove any unwanted bulk materials. Further, the CDs solution was kept at below 4 °C for further use^51^. Synthesised samples were denoted by *Prosopis juliflora* carbon dots [PJ-CDs].

### Characterization of PJ-CDs

XRD analysis of the PJ-CDs employing Cu Kα radiation (λ^1/4^ 1.54 nm) was conducted on a Philips PW 3050/10 advanced spectra were recorded at a scanning speed of 0.1 min^−1^ with 2θ angle ranging from 10° to 80°. High-resolution transmission electron microscopy experiments (HR TEM) were performed on a JEOL/JEM 2100 plus microscope (operated at 200 kV). Fourier transform infrared spectra were collected with a FTIR spectrometer (Shimadzu IR spirit) in the wavelengths range of 4000–00 cm^−1^ at a room temperature. Optical properties like UV-Vis absorbance were assessed by using UV-Visible Spectroscopy (model: UV-1800, Make: Shimadzu) and the scanning electron microscopy (SEM) (Carl Zeiss Microscopy GmbH, Germany). Fluorescent images were captured by placing in; fluorescence spectra of CDs were recorded with the aid of Fluorescence spectrophotometer, Jasco, FP-8200. (Japan make).

### Bacterial culture

In order to guarantee the sterility, all utensils were autoclaved at 121 °C for 20 min. *Staphylococcus aureus* (*S.aureus*) (ATCC25923) and *Escherichia coli* (*E.coli*) (ATCC25923) bacterial strains were procured from Department of Microbiology, Periyar University, Salem, India. Cultures were maintained at 37 °C under a shaking speed of 180 rpm in a nutrient broth medium. The load of bacteria was detected by evaluating OD at 600 nm via UV-Visible Spectroscopy. The bacterial solution was diluted to till the cell concentration of 10^6^ to10^7^ CFU/mL.

### Antibacterial activity

The antibacterial activity of the synthesized PJ-CDs against *S.aureus* and *E.coli* were evaluated using the well plate technique^52^. Petri dishes and sample were sterilized at 120 °C for 20 min before the antibacterial assay. The overnight grown bacterial culture was swabbed over the surface of the nutrient ager media using a sterile cotton swab for the even spread of the bacteria. Synthesized PJ-CDs stock solution was diluted with sterile Double Distilled Water (DDW). About 1.5 mg/mL of CDs solution was poured into the well, kept for 24 hrs at 37 °C and the inhibition zones were successively observed. The experiments were carried out in triplicate. The antibacterial activity was observed by inhibition area (mm) and compared with negative control (DDW).

### Minimal inhibitory concentration (MIC) assay

The minimum inhibitory concentration (MIC) is calculated by micro dilution 96-well cell culture plate route according standards guidelines. Each well contains 50 µl of bacteria and 50 µl of various concentrations (0.5, 0.75, 1, 1.25, 1.50, 1.75, 2 mg/mL) of synthesized PJ-CDs. The control well is bacterial culture in the sterile nutrient broth without any drug treatment. Sample loaded plate was kept in a shaking incubator at 180 rpm and 37 °C for 24 hrs, OD_600_ values were monitored by a micro-plate reader at various time durations. This experiment was carried out three times. Besides, the bacterial inhibition of 24 hrs was calculated according to the following formula^53^.

$${\text{Bacterial }}\,{\text{inhibition}} \left( \% \right) = {\text{ OD }}{\text{of}}{\text{ Control-OD}}\,{\text{ of }}{\text{Test }}{\text{Bacteria}}/{\text{OD }}{\text{of}}{\text{ Control}} \times {1}00.$$

### Bioimaging

Since the commercial florescent dyes such as propidium iodide and propidium azide have the ability to enter the cell wall of the death bacterial cells, they have generally utilised for DNA staining techniques. Due to the cost, toxic and photo-bleaching effects researchers are in search to develop such dyes with inexpensive, low toxic and high water solubility to conduct cell viability assays. Fluorescent properties of CDs can be wide range used for so many biological applications, compared to traditional organic dyes. In vivo cellular imaging studies were performed to carry out *Caenorhabditis elegans* incubated PJ-CDs 1 mg/ml for 8 hrs, followed by fluorescence images using a fluorescence microscopy.

## Results and discussion

### UV-visible spectroscopy

**Figure 1:**
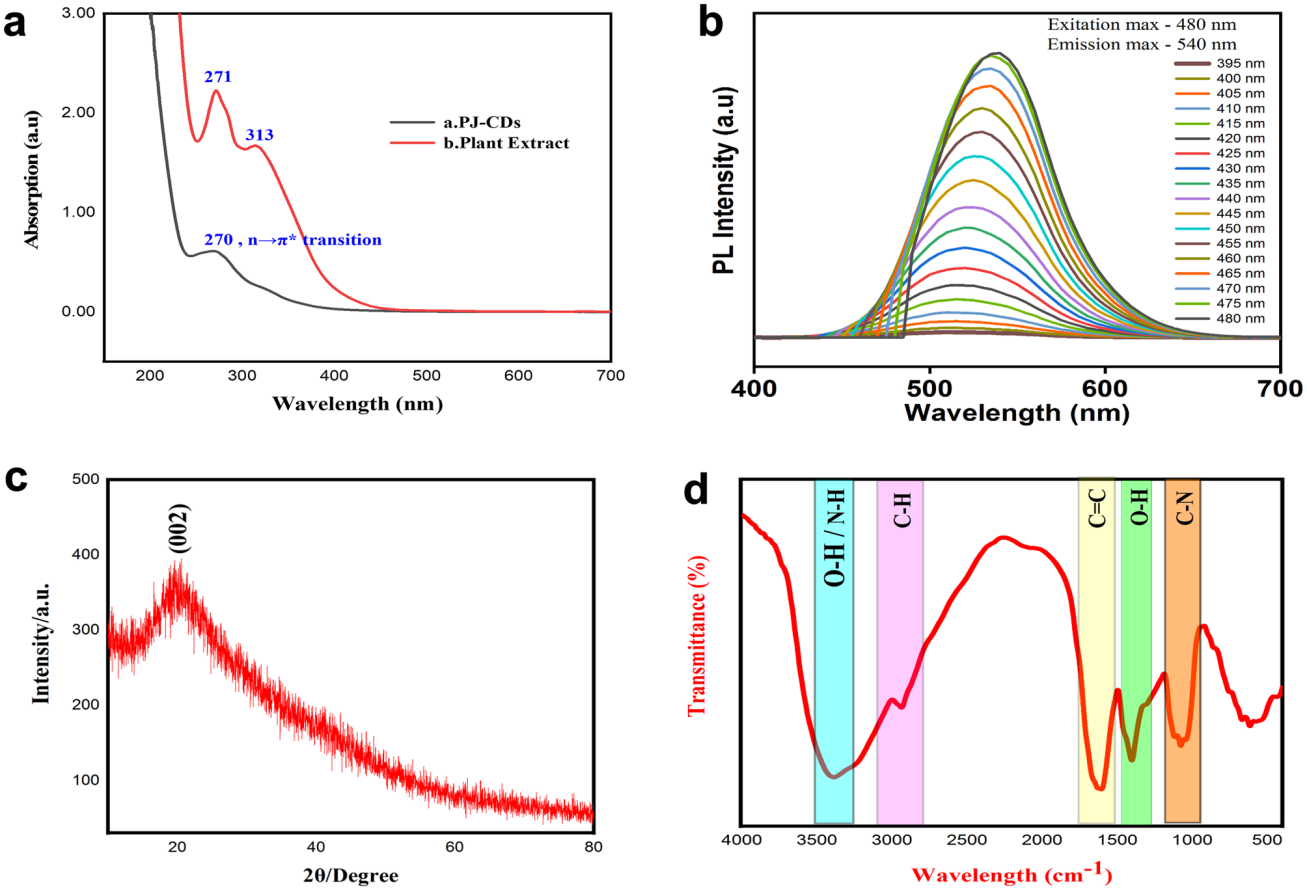
(**a**) represents the UV-visible spectra of *Prosopis juliflora* mediated carbon dots and the plant aqueous extract, (**b**) represents the Fluorescence spectrum of PJ-CDs, (**c**) shows the XRD Pattern of PJ-CDs and the (**d**) shows the FTIR spectrum of PJ-CDs synthesized from *Prosopis juliflora* leaves.

The optical characterises of the synthesized PJ-CDs sample were evaluated by UV-Vis spectrophotometer (Fig.1a,b). The UV-visible spectra of PJ-CDs (Fig. 1a) shows a peak value of 270 nm which represents the n→π* transition of carbonyl functional groups. Whereas the plant extract (Fig. 1a,b) observed with two peak at 313 and 271 nm confirms to previous report^54^. In a previous report, a water-soluble CDs produced from citric acid and Curcumin with effective antibacterial and anti - biofilm activities showed the UV-Vis spectra peak value of 280 nm which stands for n-π* transition^55^.

### Quantum yield calculation

Quantum yield of green synthesis PJ-CDs was determined by employing quine sulphate solution as a standard. Quine sulphate solution dissolved in 0.1 M of H_2_SO_4_ was used as a standard with Q Value of 54 % at 345 nm excitation wavelength for quinine sulphate. Equation is used for the evaluation of quantum yield of carbon dots^56^.

$${\text{\% Q = \% Q}}_{{{\text{Std}}}} \left( {{\text{I/I}}_{{{\text{Std}}}} } \right) \, \left( {{\text{OD}}_{{{\text{Std}}}} {\text{/OD}}} \right) \, \left( {{\raise0.7ex\hbox{${\eta^{2} }$} \!\mathord{\left/ {\vphantom {{\eta^{2} } {\eta^{2Std} }}}\right.\kern-0pt} \!\lower0.7ex\hbox{${\eta^{2Std} }$}}} \right){.}$$

Q is fluorescence quantum yield, I is the combined fluorescence intensity, OD is the UV-Vis absorbance, Ƞ- is the refractive index of the solvent for PJ-CDs suspension solution as water (ƞ = 1.33) and 0.5 M H_2_SO_4_ in water (Ƞ= 1.76).

### Photoluminescence assay

The fluorescence study was carried out in wavelength ranges between 395 and 480 nm. Figure 1b shows emission peak at 540 nm around an excitation wavelength of 480 nm. Moreover, the fluorescence intensity steadily shifted red due to the defect luminescence^40^. The absolute photoluminescence quantum yield was 7.88 %. Table 1, represents various synthesized carbon dot materials and their quantum yield compared with this present study. Two major explanations are suggested from the photoluminescence of carbon dots. First one is the CDs have a surface and edge state that explains when graphene is cutting in distinct ways, such as armchair and zigzag edges. Edges types have a larger role in the electronic properties of CDs. On the surface of carbon dots, oxygen and amine are influencing on optical band gap. As a result, the synthesis method and starting material have a significant impact on the electronic properties of Carbon dots. Second main reason is contributed to the quantum confinement in carbon dots. CDs photoluminescence origin is allocated to different reasons such as surface groups, size, defect, and passivation, and the recombination of electron hole pair located within sp^2^ carbon embedded in sp^3^ matrix^57^.

**Table 1:** Comparison of optical properties of PJ-CDs with quantum yield from different precursors and several applications.

S. no	Plant materials precursor	Quantum yield (%)	Application	Reference
1	Ginkgo fruit	3.33	Cell imaging	^58^
2	Carrot juice	5.16	Cell imaging	^59^
3	*Syzygium cumini* fruit	5.9	Fe3+ ion detection, cell imaging	^60^
4	Canon ball fruit	7.01	catalytic reduction of textile dyes, Fe3+ ion detection	^61^
5	Rice bran	7.4	Degradation of methylene blue, fluorescent ink applications	^62^
6	Waste chimney oil	7.5	Sensors, biolabeling and ink	^63^
7	*Prosopis juliflora* leaves	7.88	Antibacterial activity	Present study

### XRD

Synthesized PJ-CDs solid sample was covered on glass substrate and submitted for crystal nature characterization. The XRD results (Fig. 1c) was documented with a graph with 2θ range vs intensity at the X axis and Y axes respectively. which shows a broad peak position at 2θ = 20.74°, (JCPDS No: 82-0505). This PJ-CDs d spacing value 2.3 nm, according to the lattice plane 002, the biosynthesized PJ-CDs was calculated using Debye Scherrer’s equation (Holzwarth and Gibson 2011). Where K denotes the shape constant of the geometric factor (0.9), λ represents wavelength, β is the line broadening at half-maximum intensity, θ is the Bragg angle, and D is the particle average crystalline size of the nanoparticles (65), and appropriate lattice spacing (002). These results indicate that the nature of Carbon dots was amorphous properties. This amorphous behaviour is due to PJ-CDs possessing closely C–N, C–C and C=O bonds of CDs^64^. In another study report in fluorescent carbon dots synthesis from neera plant its detection of silver ions (XRD peak values as 2θ = 20.82°)^65^.

$${\text{D = k}}\lambda { /}\beta {\text{ 1/2 COS}}\theta {.}$$

### FTIR

The broad band absorption peaks at 3391 cm^−1^ is due to O-H stretching, 2918 cm^−1^ is due to the C–H of alkanes (Fig. 1d). The peaks at 1604 cm^−1^ and, 1400 cm^−1^ correspond to the absorption peaks attributed to C=C and O–H stretching vibrations, respectively. Additional peaks at 1079 cm^–1^ signifies C-–N stretching vibrations. The presence of the hydroxyl group (O–H) is critical in enhancing the antibacterial activity of the prepared PJ-CDs^66^.

### Scanning electron microscopy (SEM) - EDX mapping

**Figure 2:**
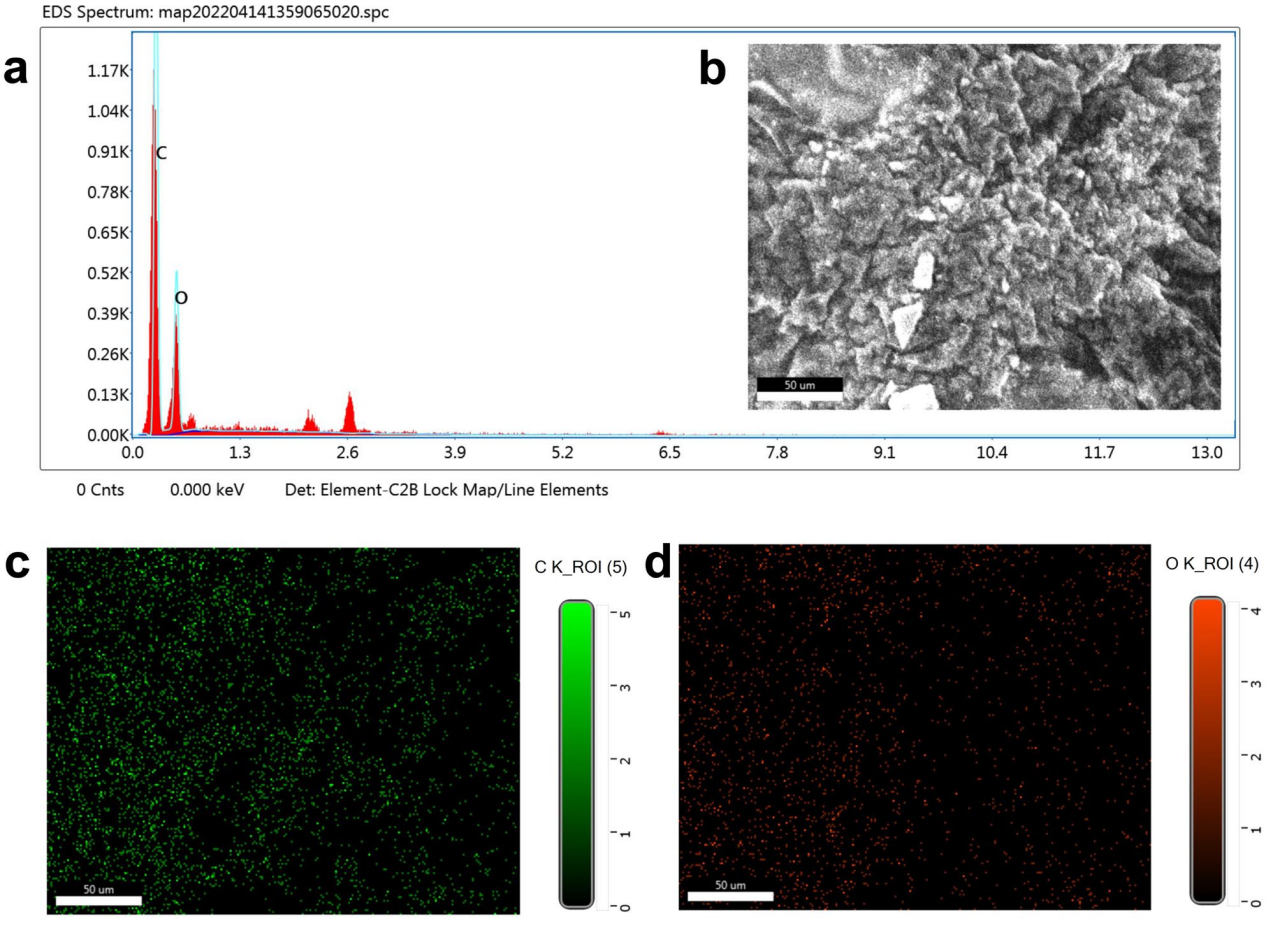
Represents SEM-EDAX mapping PJ - CDs synthesized from *Prosopis juliflora* leaves, and element mapping (**a**,**b**), carbon (**c**) and oxygen (**d**).

EDX is used to identify elemental composition of synthesized PJ-CDs. The biosynthesized PJ-CDs were observed on a copper-coated carbon grid under SEM. EDX scanning performance was done with a voltage range in 40 kV and mV with cu-k radiation in the 2θ range of 10 to 80 (Fig. 2a,b). The results showed the product was made up of two elements namely carbon level in the level of 61.08 % and oxygen level in the level of 38.92 % (Fig. 2c,d). This result confirms that the carbon is the main element in synthesized PJ-CDs. The oxygen can be related to the functional groups on the CDSs such as hydroxyl, carboxyl, alkyl groups^67^.

### HR-TEM

**Figure 3:**
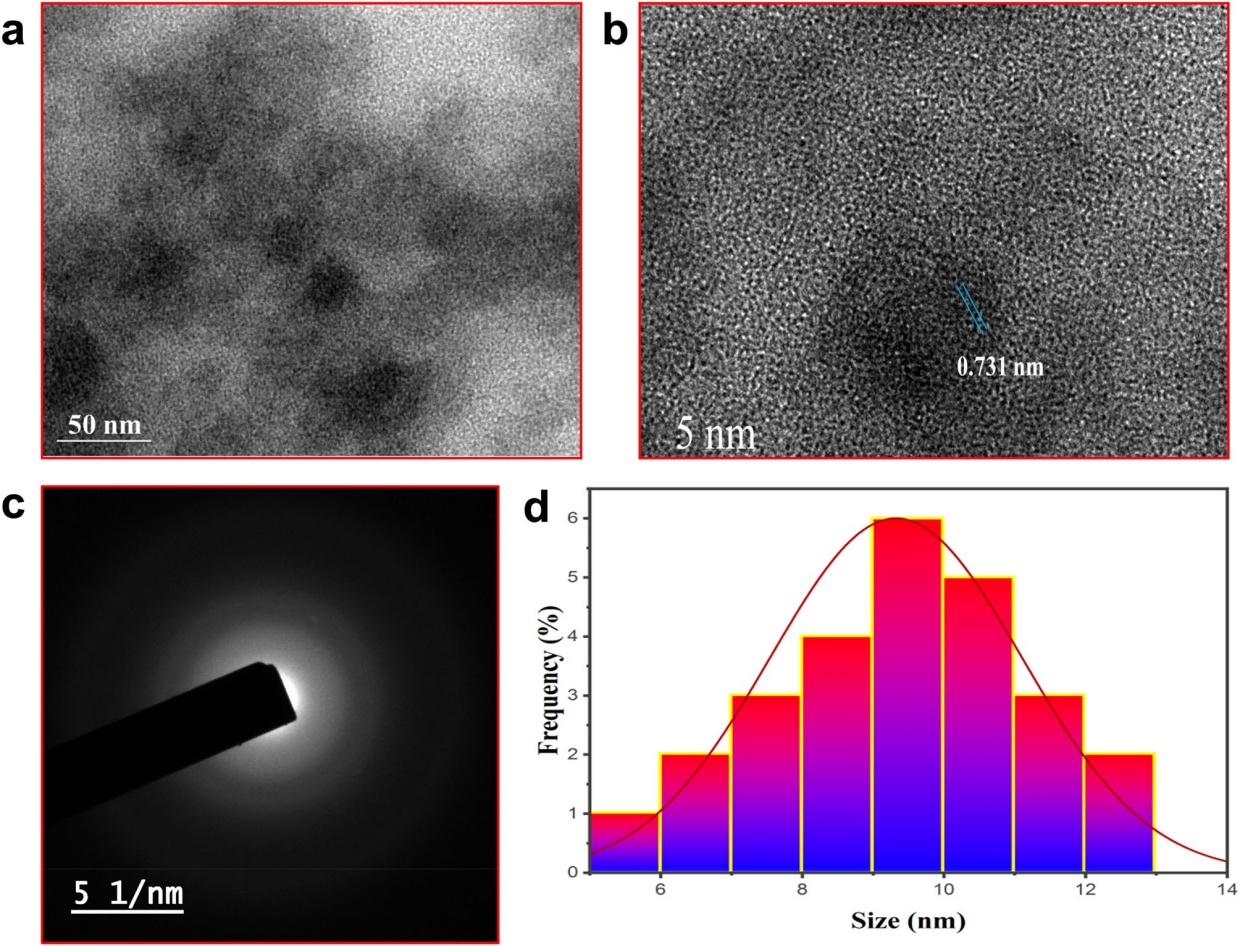
Represents HR-TEM analysis (**a**,**b**), SAED of PJ-CDs (**c**) and particle size distribution of histogram (**d**) PJ-CDs synthesized from *Prosopis juliflora* leaves.

The size and structural morphology of PJ-CDs particles were confirmed by the HR- TEM. Fig.3a indicates the particles were spherical in shape, and Fig.3b which suggests that a PJ-CDs lattice space of 0.731 nm was observed. The diffraction pattern presented (Fig. 3c) indicates the amorphous nature of the synthesized PJ-CDs^68^ and image J software was used to identify the size distribution of PJ-CDs (Fig. 3d) Which showed that the majority of PJ-CDs were in range of 5-12 nm with average size of 8 nm.

### Effects of salinity and ionic strength on fluorescence

**Figure 4:**
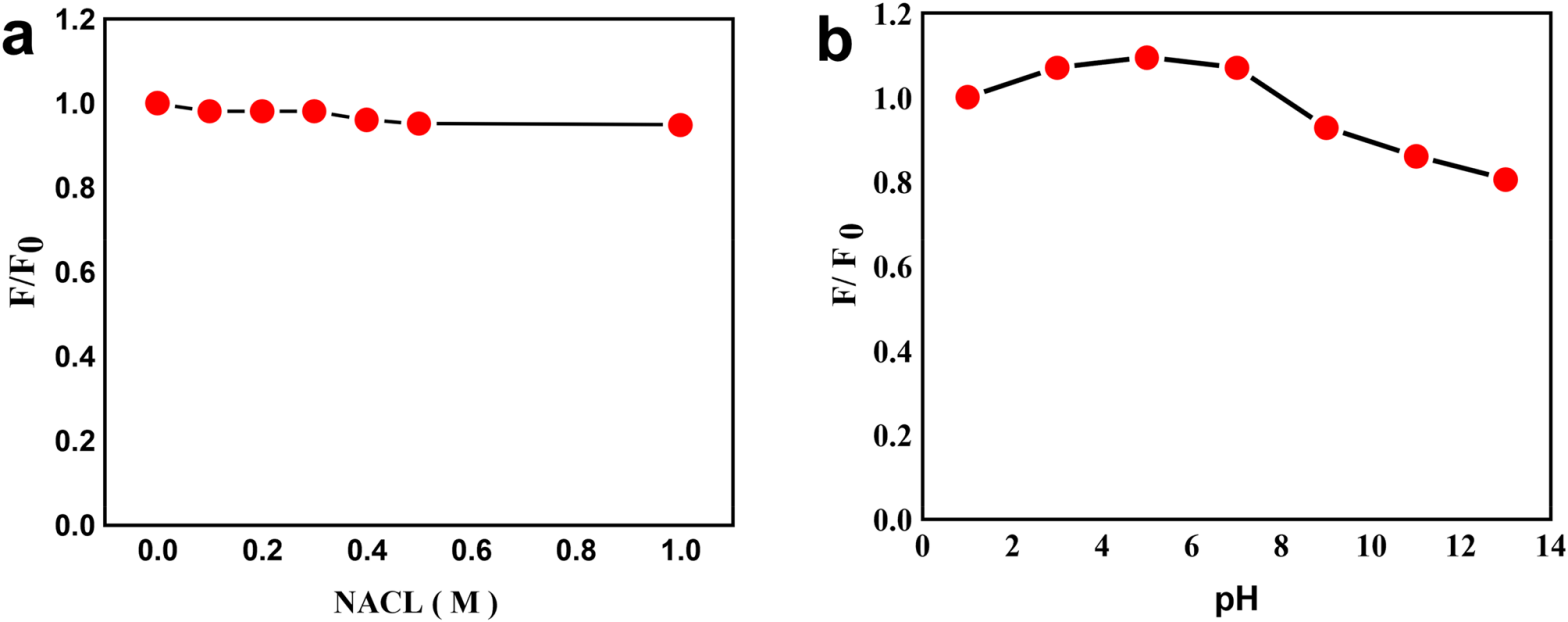
Represents fluorescence intensity of PJ-CDs at excitation wavelength of 436 nm indicate the ionic strength of NaCL solution of different concentration in 0.1M to 1M (**a**)**,** and at different pH (**b**) in PJ-CDs synthesized from *Prosopis juliflora* leaves.

Synthesized PJ-CDs fluorescent intensity was investigated at the solutions of various ionic strengths and various pH. There was no change in fluorescent intensity in various ionic strength of NaCL From 0.1 to 1 M as shows (Fig. 4a). The effect of pH on the fluorescent intensity of the CDs was investigated over the pH ranges such as 1-13 (Fig. 4b). The intensity of the PJ- CDs was affected only at acidic and alkali pH conditions. The florescence intensity of PJ-CDs depends on the pH of the solution and there was no shift in emission wavelength. Decreasing pH increased the fluorescence intensity. At the same time increasing the pH values decreased the fluorescence intensity. This study shows that at low pH values, CDs most likely exist as isolated species in the aqueous solution. Once pH increased, the CDs become agglomerated due to non-covalent molecular interaction such as hydrogen bonds between carboxyl groups^69^.

### Bacterial activity

**Figure 5:**
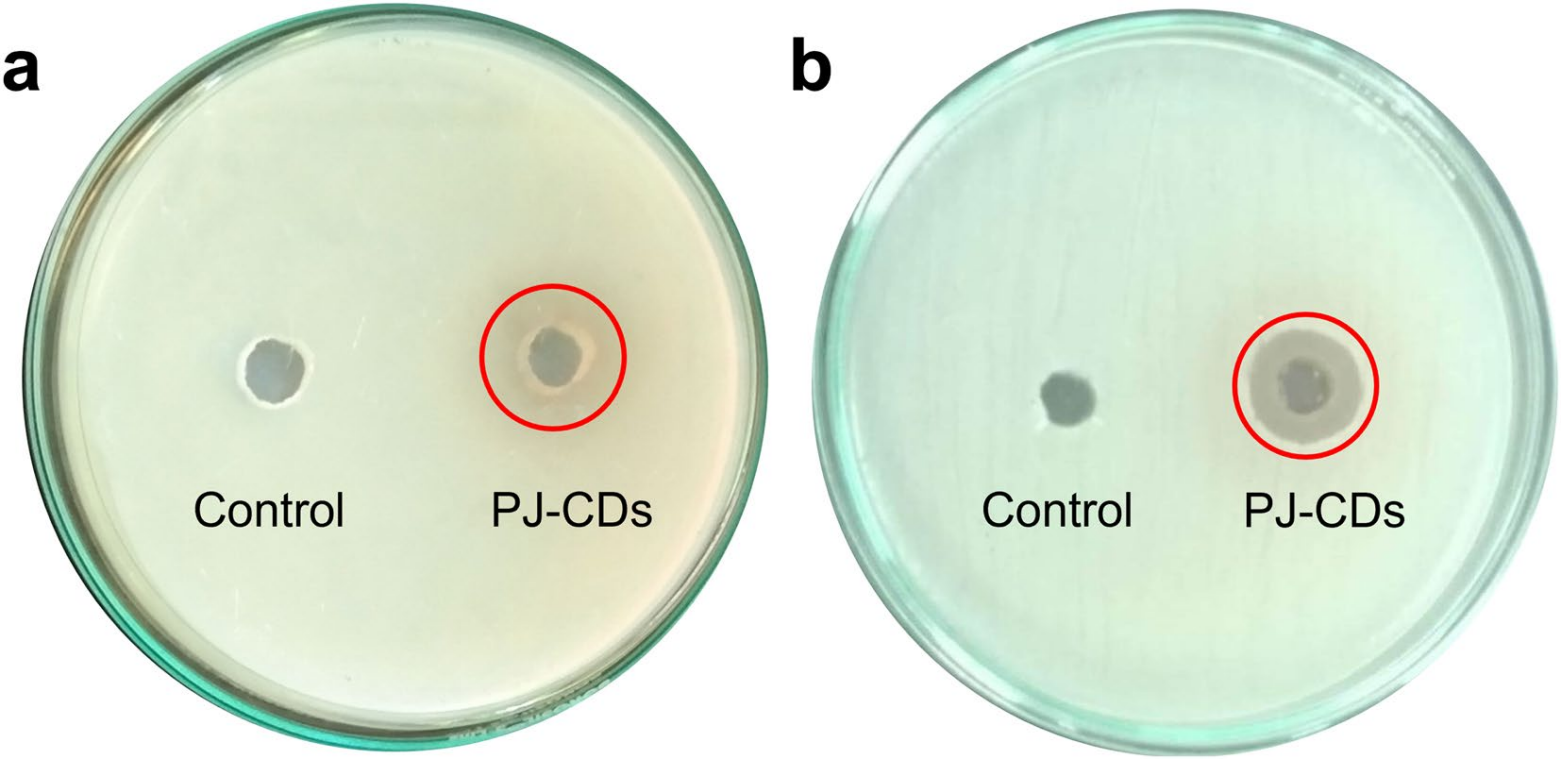
Represents the antibacterial activity results. *E. coli* found to be resistant (**a**) for the PJ-CDs, whereas, the *S. aureus* found to be sensitive (**b**).

**Figure 6:**
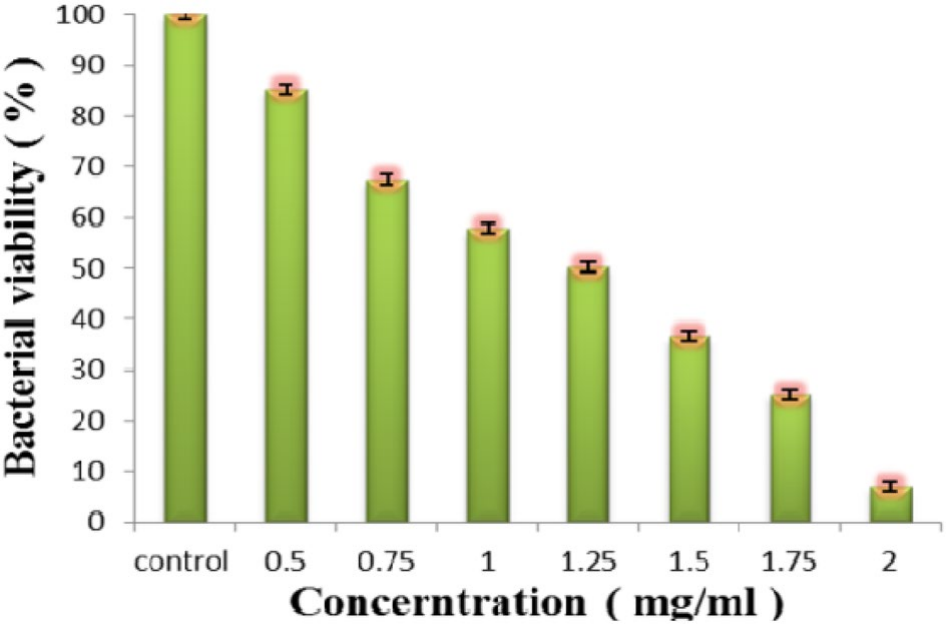
Shows the MIC of bacterial activity in *S. aureus* to various concentrations of PJ-CDs.

The synthesized PJ-CDs was studied their antibacterial activity against gram positive and gram-negative bacteria. The PJ-CDs have high ability to control gram positive bacteria *S. aureus*, growing as determined the zone of inhibition (Fig. 5b). Non-functionalized PJ-CDs revealed no antibacterial activity against *E. coli* (Fig. 5a)^8^. Table. 2 shows antibacterial activity synthesized PJ-CDs from the current investigation to other published work. Minimal inhibitory concentration is often recognised as the gold standard for evaluating material’s antibacterial properties. As shown in Fig. 6, with increase in concentration bacterial viability decreased in a dose dependent manner. The synthesized PJ-CDs have significant inhibition abilities to *S. aureus* with MIC at 1.50 mg/mL, these results are comparable with the similar studies of Chai et al.^70^who prepared P doped CQDs. The MIC of the prepared P doped CQDs against *S. aureus* was found at 1.44 mg/mL^70^. According to few studies, antibacterial CDs are only effective against gram positive bacteria and not against negative bacteria^71^.

**Table 2:** Comparison of antibacterial activity attained in current work with that of other carbon dots synthesized using plants and their secondary metabolites.

Samples	Bacterial species	Zone of inhibition (mm)	Reference
Henna CDs	*E. coli*	12	^72^
Aloe-vera conjugated CQD	*E. coli*	19	^73^
*Prosopis juliflora* leaves	*E. coli*	0	Present study
Henna CDs	*S. aureus*	17	^72^
Aloe-vera conjugated CQD	*S. aureus*	12	^72^
Curcumin QDs	*S. aureus*	11.3	^74^
Lys-CQDs	*S. aureus*	16	^75^
Aloe-vera conjugated CQD	*B. subtilis*	15	^73^
*Prosopis juliflora* leaf	*S. aureus*	18	Present study

### Antibacterial activity mechanisms of the PJ-CDs

The large π- conjugated carbon quantum dots can easily be bind to the bacterial cell wall through the electron. In general, for any antimicrobial activity the initial step is a physical contact with cells through either electrostatic action or chemical conjugation. The antibacterial activity will be based on electrostatic interactions, reactive oxygen species generation (ROS) and light irradiation. Especially the reactive oxygen species generation is the most important antibacterial activity mechanism among them. The antibacterial activity may be attributed to several functional groups which present in the green synthesized PJ-CDs that interfere with cellular enzyme roles and inhibit the cellular proliferation^76^. The FTIR results elements that possess positive charges that linked with negative charges microbe ultimately found in death of microorganisms. CDs not only are effectively ingested into bacteria, but also spread into bacteria by diffusion. When CDs enter into the bacterial cell, they will accumulate in DNA/RNA and affect their structures which cause the DNA double helix to separate. Moreover, the CDs can form a covering on the surface of bacteria, and finally lead to cell death^40^.

### Bioimaging applications

**Figure 7:**
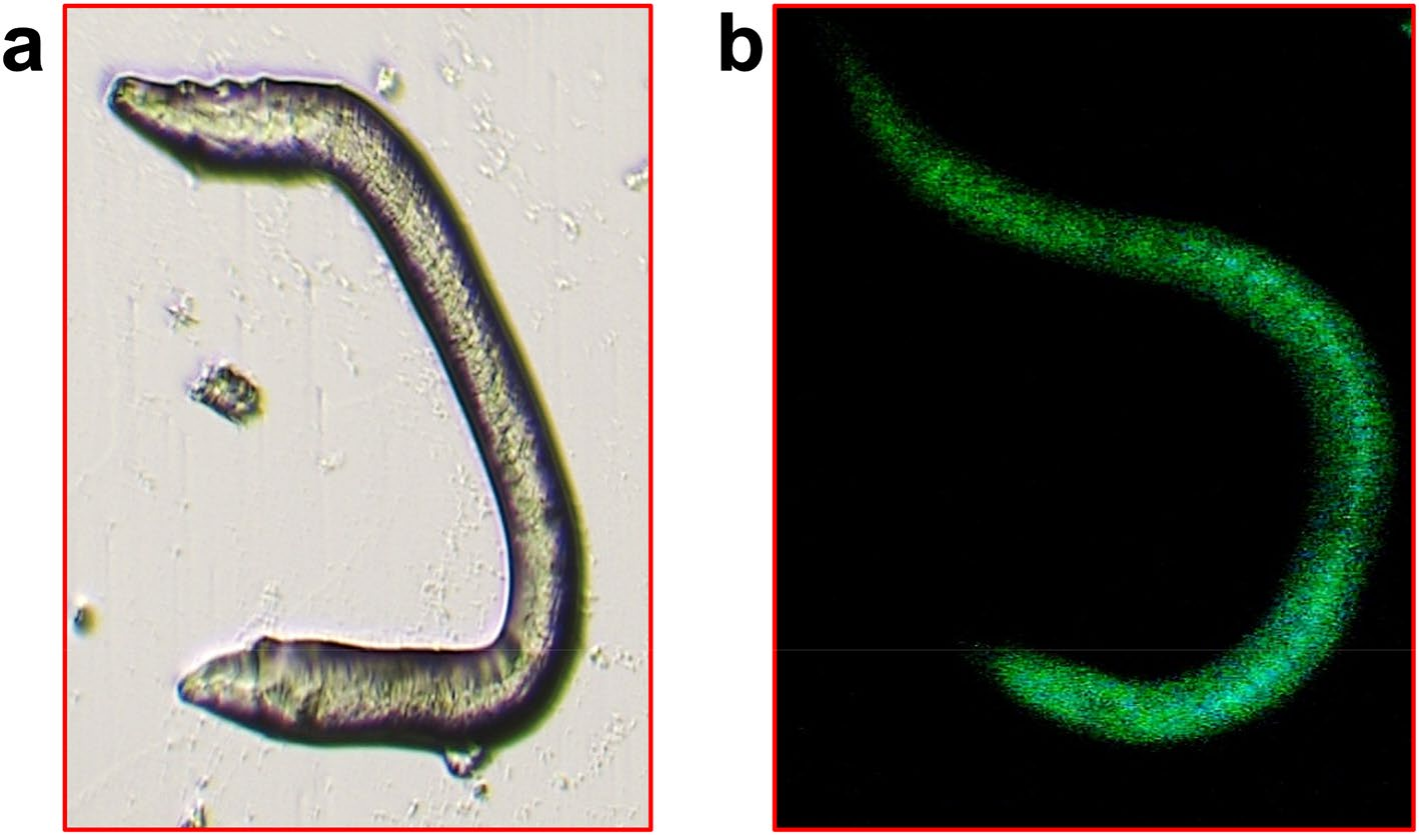
Shows the fluorescence imaging of *C. elegans* treated with PJ-CDs synthesized from *Prosopis juliflora* leaves at excitation wavelength (**a**) bright field (**b**) 470 nm.

*Caenorhabditis elegans* emitted green excitation at a laser excitation of 470 nm (Fig. 7). PJ-CDs have low toxicity, excellent bio-compatility and fluorescence emitting properties. In this study we demonstrated the synthesized PJ-CDs entry into the body of *Caenorhabditis elegans*. The bioimage study of synthesized PJ-CDs has shown that they have not considerably reduced the *Caenorhabditis elegans* cell viability even at 1 mg/mL, and also observed that here were no morphological changes in cells after incubation with PJ-CDs. Due to their photo-stability, water stability, bioimaging effects it is conformed that the synthesized PJ-CDs were more suitable for biocompatibility and hence are suitable for biomedical and other biological applications. The CQDs isolated from banana peel waste materials by hydrothermal technique indicated a bioimage of nematodes was reported by Atchudan et al.^77^. As a result, quantum confinement, surface traps, aromatic structure creation, and exciton recombination have been suggested as the involving mechanisms^78,79^.

## Conclusion

The multifunctional PJ-CDs were synthesized from natural source *Prosopis juliflora* leaves without the influence of any hazardous and cost-effective chemicals by an eco-friendly hydrothermal technique with a quantum yield of 7.88 %. We propose a green, cost-effective, environmentally friendly and perfectly sustainable large scale production method for CD synthesis. *Prosopis juliflora* leafs are one of the easily and enormously available cheap biomass. Hence conversion of such biomass into valuable products such as PJ-CDs is of great interest. They have various organic phytochemicals that can serve as an effective carbon source for preparing CDs. The average particle size of the PJ-CDs was 8 nm with a spherical shape which was confirmed by HR-TEM. The XRD analysis confirmed the amorphous graphite carbon structure of the PJ-CDs. FTIR indicates that the presence of hydrophilic groups (-OH, and -COOH) have led to greater water-soluble properties. The antibacterial potentially PJ-CDs can also be useful in providing sterile environment for bio-image and biomedical application.

## Data Availability

The data analysed during the current study are not publicly available due the project regulations, but are available from the corresponding author on reasonable request.
